# Age-Related Increase in Electromyography Burst Activity in Males and Females

**DOI:** 10.1155/2013/720246

**Published:** 2013-07-31

**Authors:** Olga Theou, Darl Edwards, Gareth R. Jones, Jennifer M. Jakobi

**Affiliations:** ^1^Geriatric Medicine, Dalhousie University, 5955 Veterans' Memorial Lane, Halifax, NS, Canada B3H 2E1; ^2^Medicine, University of Toronto, Toronto, ON, Canada M5S 1A8; ^3^Health and Exercise Sciences, University of British Columbia Okanagan, 3333 University Way, Kelowna, BC, Canada V1V 1V7

## Abstract

The rapid advancement of electromyography (EMG) technology facilitates measurement of muscle activity outside the laboratory during daily life. The purpose of this study was to determine whether bursts in EMG recorded over a typical 8-hour day differed between young and old males and females. Muscle activity was recorded from biceps brachii, triceps brachii, vastus lateralis, and biceps femoris of 16 young and 15 old adults using portable surface EMG. Old muscles were active 16–27% of the time compared to 5–9% in young muscles. The number of bursts was greater in old than young adults and in females compared to males. Burst percentage and mean amplitude were greater in the flexor muscles compared with the extensor muscles. The greater burst activity in old adults coupled with the unique activity patterns across muscles in males and females provides further understanding of how changes in neuromuscular activity effects age-related functional decline between the sexes.

## 1. Introduction

Accelerometers, pedometers, and global position system (GPS) have become familiar tools for assessing age-related changes in mobility and physical activity during daily life [1, 2], but these devices provide no indication of muscle activity governing movement. Laboratory studies using electromyography (EMG) have provided considerable understanding of age-related changes in muscle activity for simple isometric and anisometric contractions [3–5], gait [6], and discrete tasks [7] executed under experimental constraints. With the advancement of technology, portable EMG devices now offer a means to characterize muscle activity (bursts) and quiescence (gaps) over long durations outside the laboratory, enabling discrete measurement of neuromuscular control within the environmental context of daily life [8, 9]. 

 Quantification of gaps in EMG for a discrete task and during daily life activities, in young and old adults, suggests muscle quiescence decreases in old adults and is less in women compared with men [10]. For a discrete task when the EMG is quantified through bursts, muscle activity increases in old adults relative to young adults and is also greater in women [8, 11]. Together, measurement of bursts and gaps for a discrete task, such as carrying a bag of groceries, suggests that muscles are active more often and “rest” less in older adults and women. Age-related remodeling [12, 13], decreased muscle strength [3, 14, 15], and greater declines in performance for fast velocity relative to slow velocity movements [16, 17] likely predispose old adults to increased bursts to execute activities of daily life. Similarly, reduced muscle mass and quality have been observed in upper and lower limbs of women and may necessitate a higher level of muscle activity to execute daily life [18, 19]. Quantification of burst activity during daily life will provide insight into muscle activation required for functional movement executed within the lived environment beyond the confines of a controlled laboratory setting where factors such as stress and fatigue are not encompassed within a discrete task.

 In addition to the increase in burst activity in the arm and thigh muscles of old adults for a discrete task [10, 11], prior studies suggest that the duration of burst activity was longer in females than males in the biceps brachii of young adults [20]. Muscle-dependent differences should not be surprising during daily life as laboratory-based motor unit and surface EMG measures describe muscle specific age-related decline in motor unit activity [3, 21] and sex-specific decline in force control [22]. Thus, to better understand whether muscle activity increases with age during daily life and determine sex-specific changes in functional muscle pairs, assessment of EMG in young and old males and females needs to be undertaken during a typical day. The purpose of this study was to quantify age- and sex-related alterations in burst activity in young and old males and females in lower limb and upper limb muscles over the course of a weekday. It was hypothesized that burst activity would be greater in old adults relative to young adults due to age-related changes in neuromuscular properties. In addition, burst activity would be exacerbated in females and in flexor muscles consistent with a previous report in young adults [20].

## 2. Methods

 Thirty-one healthy adults from the general populous of greater Windsor-Essex County of South Western Ontario participated in this study. All subjects were free of known neurological or neuromuscular conditions. Individuals were excluded if they participated in structured regular exercise training programs greater than three times per week. Volunteers were divided into four groups based upon age and sex. Age criterion for the young adults was less than 26 years whereas the minimum age for the old adults was 70 years. The local Human Research Ethics Board approved this experiment, and informed written consent was obtained prior to participation.

 Subjects participated in one 8-hour recording day and visited the laboratory twice on the same day. The first session was in the morning, approximately 90 minutes after the subjects awoke. The Yale Physical Activity Survey [23] was administered to assess differences in energy expenditure and physical activities between age and sex. Subsequently, electrodes were placed on the biceps brachii (BB), triceps brachii (TB), vastus lateralis (VL), and biceps femoris (BF), and maximum voluntary exertions (MVEs) were performed for the four muscles to record maximal electromyography (EMG) for normalization of surface recordings. Following the initial MVE assessment, subjects left the laboratory and were asked to partake in normal daily activities, defined as events that were typically and regularly performed. Subjects were asked not to engage in any water activities (i.e., swimming, showering). After eight hours, subjects returned to the laboratory and the MVEs were repeated to verify the integrity of the recording off-line. Subsequently, the EMG equipment was removed and subjects were debriefed. 

### 2.1. Electromyography

 Long-term EMG was recorded using surface electrodes (20 mm interelectrode distance) placed on the long head of the BB and the lateral head of the TB as well as the VL and BF as previously described [8–11, 24, 25]. BB and TB are the principle elbow flexors and extensors, respectively, and are the primary contributors to reaching in order to execute functional upper limb movements. VL and BF are large muscle groups utilized to transport the body during upright posture and gait and exert flexion and extension force about the knee and undergo severe age-related weakening which is related to functional decline. Electrodes were attached to the skin using double-sided medical grade die cut application tape [26]. The DataLOG unit (Biometrics P3X8, Gwent, UK; 9.5 × 15.8 × 3.3 cm, 380 gram) which recorded signals was attached to a belt and worn on the hip. The electrodes and device did not impair or limit movement or activities of daily living.

### 2.2. Maximum Voluntary Exertions

 MVEs were performed three times for each muscle (VL, BF, BB, and TB) both in the morning and afternoon, as previously described [11, 24]. Briefly, for the arm muscles the MVEs were performed with the elbow joint at 90°. The experimenter instructed and resisted a maximum pull-up (flex arm) and push-down effort (extend arm) for approximately 3 seconds to assess maximum BB and TB activities, respectively. For the two lower limb muscles, the subject was seated in a rigid chair against a wall that was adjusted to the subject's height so that the knee and hip joint could be positioned to 90° for the maximum flexion/extension efforts of the leg that were executed against experimenter resistance. A canvass strap was fixed around, the waist of the subject and the chair to support consistent positioning. All subjects were highly encouraged with verbal instruction prior to the MVE and subsequent to the attempt to exert the greatest possible effort. When aberrations in position occurred the subject was informed of the deviations and attempted the MVE effort again. The EMG value derived from the MVE was a computed rectified and integrated value recorded from the middle of the MVE contraction.

### 2.3. Signal Processing and Data Analysis

 All EMG signals recorded during the MVE and the 8 hours testing were amplified (1000x), filtered (20–450 Hz), and stored on the 512 MB MMC flashcard at a sampling rate of 100 Hz for subsequent analysis. Data from the flashcard was transferred to the computer hard drive via USB cable and imported into the Biometrics (Gwent, UK) program. From the Biometrics (Gwent, UK) program, data was exported to Spike 2 version 5 (Cambridge Electronics Design, Cambridge, UK) for further processing in a custom analysis script. All signals were rectified, smoothed (0.01 sec), and down-sampled (100x) to 10 Hz prior to analysis. In some instances, artifacts due to electrode or device contact occurred, and these sections were time-locked and deleted across all channels simultaneously. These artifacts were limited and their occurrence was similar between age and sex groups. Data artifact was identified through visual inspection which was then confirmed by taking the root mean square (RMS) amplitude of the waveform in 0.1-second increments and identifying plateaus (square waves) in the recording. A custom script was utilized within Spike 2 for the burst analysis. An EMG burst was defined as any interval in the recording that had an amplitude >2% of the maximum EMG value and duration >0.1 seconds. The EMG data was analyzed and quantified using five burst characteristics: number of bursts, mean duration (seconds), burst percentage (% of total recording time occupied by bursts), peak amplitude (the peak amplitude of bursts averaged across all bursts, %MVE), and mean amplitude (average of the mean amplitude of all bursts, %MVE). 

### 2.4. Statistical Analysis

The Statistical Package for the Social Sciences (SPSS, Chicago, IL, USA) for windows version 17.0 was used for statistical analysis. A 2 × 2 analysis of variance (ANOVA), with age and sex as between-subject factors, was conducted on results from the Yale Physical Activity Survey. A paired *t*-test was utilized to compare the morning and evening values for the maximum and average MVEs from each session. The five dependent variables of the burst characteristics were compared with a 2 × 2 × 4 multivariate ANOVA for the between-subject factors of age (young, old) and sex (male, female) and the within-subject factor of muscle (VL, BF, BB, and TB). The probability level for all statistical measures was set at 0.05.

## 3. Results

 The eight young males were 24.0 ± 2.5 years (90.6 ± 13.1 kg, 182.0 ± 9.4 cm) and the eight young females were 22.0 ± 1.6 years (57.4 ± 8.5 kg, 162.6 ± 4.4 cm). The eight old males were 76.6 ± 2.8 years (83.9 ± 4.0 kg, 172.3 ± 4.9 cm) and the seven old females were 77.3 ± 4.5 years (74.3 ± 21.8 kg, 159.2 ± 5.9 cm). Total physical activity did not differ between age groups (young 8032 ± 2386 kcal/week; old 7532 ± 4975 kcal/week; *P* = 0.72) or sexes (male 7788 ± 4637 kcal/week; female 7792 ± 2819 kcal/week; *P* = 0.99). 

The maximal and average MVE values from the morning and evening sessions did not differ; thus, the highest MVE recorded from the six attempts was utilized for normalization of the long-term EMG data and determination of burst characteristics. The three-way interaction between age, sex, and muscle was significant (*P* = 0.04). In order to identify the contribution of individual dependent variables to this significant interaction of the multivariate ANOVA, a 2 × 2 × 4 (age × sex × muscle) univariate ANOVA was utilized for each burst variable. 

For the number of bursts, mean amplitude, and burst percentage, there was no three-way interaction (age × sex × muscle; *P* = 0.06). The 2 × 2 (age × sex) interactions were not significant for these variables (*P* > 0.05); however, there was a 2 × 4 (sex × muscle) interaction for burst percentage (*P* = 0.01) and a 2 × 4 (age × muscle) interaction for the number of bursts (*P* = 0.04). All three of these variables were greater (*P* < 0.05) in old adults compared with young adults (Table 1), and females had greater (*P* = 0.04) number of bursts compared with males. There was no sex effect on mean amplitude (*P* = 0.77) and burst percentage (*P* > 0.05) across muscles (Table 2). 

For burst duration and peak amplitude, significant three-way interactions were observed (*P* < 0.05). These interactions were examined further by performing a 2 × 2 univariate ANOVA (age × sex) for each muscle group separately and a 2 × 4 univariate ANOVA (sex × muscle) for young and old. The 2 × 2 (age × sex) interactions were significant for burst duration in the TB (*P* = 0.02) and peak amplitude in the BF (*P* = 0.01). The 2 × 4 (sex × muscle) interactions were observed for burst duration and peak amplitude of the old adults (*P* < 0.05). Burst duration was greater in old adults compared with young adults in all muscles except the TB of males (*P* = 0.92) (Table 1). Burst duration in the BB of both young and old adults (*P* = 0.01) and in the TB of old adults (*P* = 0.03) were greater in females than males. Peak amplitude in the BF of old adults was greater (*P* = 0.03) in males (Table 2).

**Figure 1:**
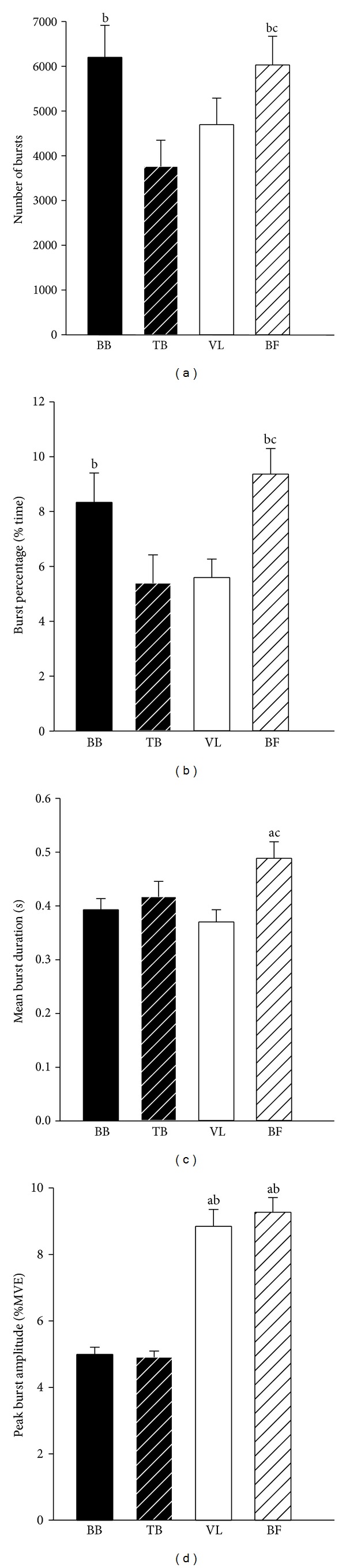
Muscle effect on burst characteristics for young adults. (a) Number of bursts; (b) burst percentage; (c) mean burst duration; (d) peak burst amplitude. BB: biceps brachii; TB: triceps brachii; VL: vastus lateralis; BF: biceps femoris; sec: seconds; %: percentage; MVE: maximal voluntary exertion. ^a^Significantly different from BB; ^b^significantly different from TB; ^c^significantly different from VL.

**Figure 2:**
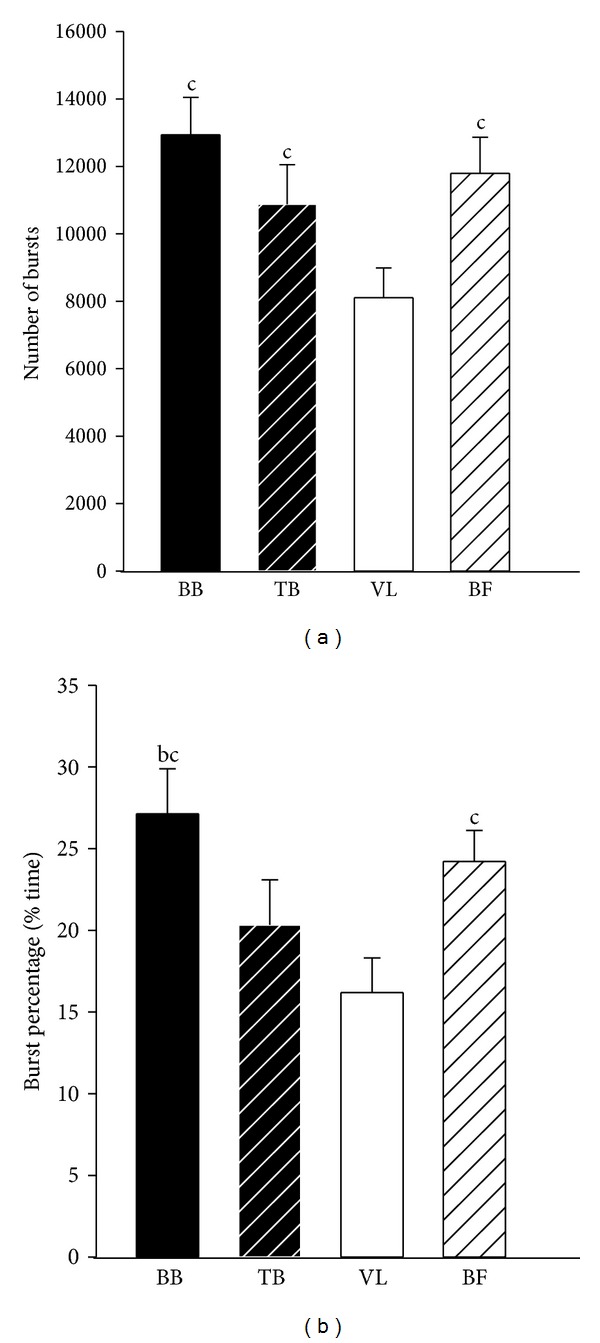
Muscle effect on number of bursts and burst percentage for old adults. (a) Number of bursts; (b) burst percentage. BB: biceps brachii; TB: triceps brachii; VL: vastus lateralis; BF: biceps femoris; %: percentage. ^a^Significantly different from TB; ^b^significantly different from VL.

**Figure 3:**
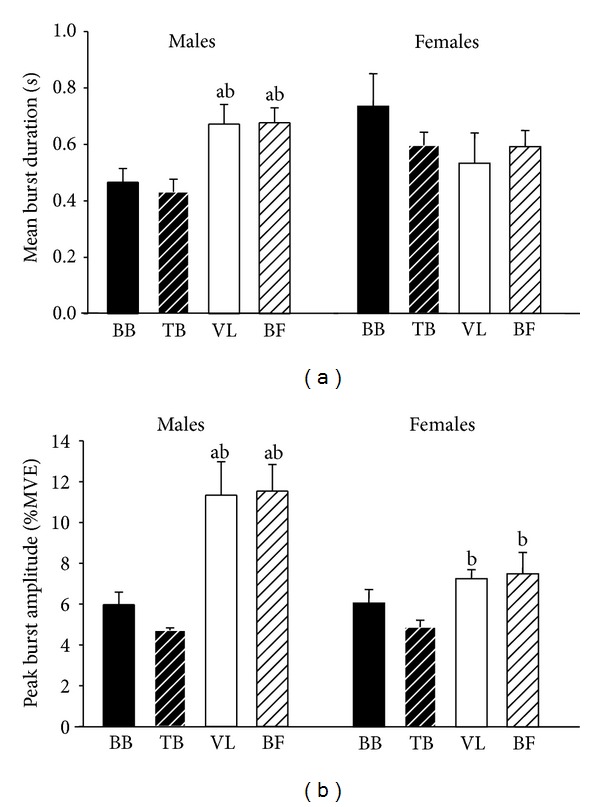
Muscle effect on mean burst duration and peak burst amplitude for old adults. (a) Mean burst duration; (b) peak burst amplitude. BB: biceps brachii; TB: triceps brachii; VL: vastus lateralis; BF: biceps femoris; sec: seconds; %: percentage; MVE: maximal voluntary exertion. ^a^Significantly different from BB; ^b^significantly different from TB.

Significant differences were found between muscles for all burst characteristics except for burst duration in old females (*P* = 0.49). In young adults, the number of bursts and burst percentage were greater (*P* < 0.01) in the flexor muscles compared with the extensor muscles in both arm (BB > TB) and thigh muscles (BF > VL). In addition, the number of bursts and burst percentage were greater (*P* = 0.01) in BF compared with TB (Figures 1(a) and 1(b)). Burst duration in BF of young adults was longer (*P* < 0.01) than the BB and VL (Figure 1(c)), and peak burst amplitude in the thigh muscles (VL and BF) was greater compared with the arm muscles (BB and TB) (*P* < 0.001) (Figure 1(d)). In old adults, the number of bursts was less (*P* < 0.01) in the VL than the other three muscles (Figure 2(a)). Burst percentage in old adults was greater (*P* < 0.05) in flexor muscles compared with extensor muscles in both arm (BB > TB) and thigh muscles (BF > VL). In addition, burst percentage was greater (*P* = 0.01) in BB compared with VL (Figure 2(b)). In old males, burst duration and peak amplitude were greater (*P* < 0.05) in thigh muscles (VL and BF) compared with the arm muscles (BB and TB) (Figures 3(a) and 3(b)). Peak burst amplitude in old females was greater (*P* < 0.05) in the thigh muscles (VL and BF) compared with the TB (Figure 3(b)). Mean burst amplitude in both young and old adults was greater (*P* < 0.05) in BF compared with the other three muscles and in BB compared with TB. 

## 4. Discussion

 The purpose of this study was to quantify age- and sex-related differences in burst activity measured with portable EMG devices during the course of a day. The key finding in this investigation was the age-related increase in muscle activation where the number of bursts was greater in females than males and flexor muscles were more active than extensor muscles. Measuring muscle activity that governs movement during daily life with portable EMG offers an early indication of the physiological alterations in muscle that manifest prior to overt declines in activities of daily living that are required for independent living. 

 The most striking difference in this study was the age-related increase in burst percentage (~190–270%Δ) with young muscles being active 5–9% of the time whereas older muscles were active 16–27%. These data clearly indicate that the manner in which daily tasks are accomplished differs at the level of the muscle, and this can be captured by long-term EMG. The observed differences between young and old adults likely arise from age-related change in neuromuscular function as physical activity was similar between these groups of adults that were separated by >50 years in age. Differences in burst activity are unlikely to be related to tasks undertaken during the day. Although daily tasks were not monitored, because subjects were free to undertake normal activities without bias of an observer, our previous work has shown that burst activity is greater in old adults when the task is matched between age groups [11]. Thus, the difference in burst activity during daily life supports our prior findings from discrete tasks [11]. Muscle weakness in old adults is a contributing factor in the age-related increase in muscle activity as laboratory-based studies report greater muscle activity in weaker individuals when the relative load for isolated contractions is matched [11]. Because old adults are typically weaker than young adults [3, 27], strength might contribute to the age-related increase in muscle activity observed for a typical day; however, strength cannot be the sole factor. Burst duration was similar between males and females in the thigh muscles, yet men are stronger than women [27]. This highlights the need to acknowledge that strength occupies a role in muscle activity that necessitates success in daily activities, but it is not the singular contributor to age- and sex-related increase in muscle activity. 

The decrease in motor unit number with increased age coupled with the remaining pool of motor units becoming less heterogeneous and larger in individual size (innervation ratio) may contribute to the age-related augmentation in burst activity. There is a positive relationship between the size of a motor unit and the resultant amplitude of EMG signal [28]; therefore, the older and larger motor units would likely have higher burst activity. This could be detected as greater mean burst amplitude in old adults compared with young adults during daily life. With increased age, movement times slow in old adults compared with young adults for a variety of tasks, and fast velocity movements are more affected by aging than slow velocity movements [16, 17, 29]. Increased movement time would likely augment that the amount of time force is maintained during execution of activities of daily life and in turn merge as greater total and mean burst duration. This increase in burst duration may allow for an enhanced level of force production to execute tasks of daily life. However, the increase in EMG burst activity during daily life, which likely facilitates the production of movement, could predispose older adults to an earlier onset of fatigue, and this may, over time, contribute to reduced fine motor control of everyday tasks similar to the decreased ability of older adults to produce steady contractions in controlled laboratory-based studies [4].

The number of bursts in females was 13–56% greater than males for both young and old adults. Increased bursts in females compared with males may be due to intrinsic properties of muscle. Studies suggest that fiber area, number, and force per cross-sectional area are reduced in females compared with males [19], and in animal models the innervation ratio is estimated to be greater in males compared to females [30]. These anatomical features coupled with a longer electromechanical delay [31], slower rate of force development [32], and slow reciprocal activation in triphasic EMG burst patterns in females compared with males [33] cause differential burst activity between sexes. These physiological and anatomical differences that culminate in greater burst activity in females are not unlike the age-related adaptations. 

 The age-related increase in burst duration of the TB was only evident in females. The sex-related differences in burst duration of the TB as well as the peak burst amplitude of the BF were only evident in old adults. In addition, muscle differences in burst duration and peak amplitude were similar between young males and females but not between old males and females. Thus, muscles of females are predisposed to alterations that culminate in earlier age-related functional decline relative to males; these changes are detectable through daily EMG. This tool offers a means for sex-specific detection of altered muscle activity with age which may enable earlier identification of impending functional changes that may have a significant impact upon physical independence [24]. Females are more likely to become frail and experience functional decline and/or loss of independence [34, 35]. 

 Differences in burst characteristics between flexor and extensor muscles were found in both the arm and thigh muscles. Flexor muscles were active for a longer duration than the extensor muscles (35–66%Δ). The greater burst characteristics of the flexor muscles compared with the extensor muscles in old adults might be related to greater age-related decline in strength and motor unit discharge rates of the flexor muscles [3, 14, 15]. Thus, the flexor muscles of the old adults need to function at a higher relative level because they have undergone greater age-related decline relative to the extensors. Therefore, this augmentation in flexor activity relative to the extensor is in part due to movements undertaken to execute daily life (self-care and feeding) which largely involve the flexors as the agonist which are reported to undergo greater age-related change [3]. 

## 5. Conclusions

 In summary, there are definitive age differences with respect to burst activity over an 8-hour recording. The muscles of old adults burst more, with higher mean amplitudes and for longer duration throughout the day and muscle of females burst more and longer than males. Overall burst activity in the flexor muscles is greater than the extensor muscles. In addition, muscle differences are consistent between young males and females but not between old males and females. Differences in muscle activity for a typical day quantified as bursts in EMG highlight alterations in neuromuscular function with age that are sensitive to sex and specific to muscle. Assessment of long-term EMG, recorded with portable devices, can be an effective way to identify physiological change prior to functional loss. This offers an effective tool for diagnosing impending physical decline in older adults through earlier identification of changes in muscle activity prior to overt functional loss. 

## Figures and Tables

**Table 1:** Age-related differences in burst characteristics for each muscle group.

	Young	Old
Number		
BB	6200 ± 3112	12935 ± 4142*
TB	3763 ± 2382	10700 ± 5167*
VL	4698 ± 2280	8048 ± 3395*
BF	6030 ± 2504	11676 ± 4391*
Duration (sec)		
BB	0.4 ± 0.1	0.6 ± 0.3*
TB^†^	0.4 ± 0.1	0.6 ± 0.1*
VL	0.4 ± 0.1	0.6 ± 0.2*
BF	0.5 ± 0.1	0.6 ± 0.2*
Peak amplitude (% MVE)		
BB	5.0 ± 0.8	6.0 ± 1.7
TB	4.9 ± 0.7	4.8 ± 0.6
VL	8.8 ± 2.0	9.4 ± 4.0
BF	9.3 ± 1.8	9.6 ± 3.8
Mean amplitude (% MVE)		
BB	0.6 ± 0.3	1.9 ± 1.1*
TB	0.6 ± 0.4	1.5 ± 0.8*
VL	0.8 ± 1.0	1.4 ± 0.9*
BF	1.2 ± 0.9	2.2 ± 1.4*
Percentage (% time)		
BB	8.3 ± 5.0	26.8 ± 11.8*
TB	5.4 ± 4.0	19.9 ± 12.5*
VL	5.6 ± 2.6	16.2 ± 8.0*
BF	9.3 ± 3.8	24.1 ± 7.3*

BB: biceps brachii; TB: triceps brachii; VL: vastus lateralis; BF: biceps femoris; sec: seconds; %: percentage; MVE: maximal voluntary exertion.

*Significantly different from young adults.

^†^Only females.

**Table 2:** Sex-related differences in burst characteristics for each muscle group.

	Male	Female
Number		
BB	8887 ± 5398	10069 ± 4541*
TB	5609 ± 4206	8731 ± 5919*
VL	5886 ± 2642	6781 ± 3922*
BF	7869 ± 4334	9713 ± 4650*
Duration (sec)		
BB	0.4 ± 0.1	0.6 ± 0.3*
TB^†^	0.4 ± 0.1	0.6 ± 0.1*
VL	0.5 ± 0.2	0.4 ± 0.2
BF	0.6 ± 0.2	0.6 ± 0.1
Peak amplitude (% MVE)		
BB	5.4 ± 1.5	5.6 ± 1.4
TB	4.8 ± 0.6	4.9 ± 0.8
VL	10.1 ± 3.6	8.1 ± 1.9
BF^†^	11.5 ± 3.7	7.5 ± 2.8*
Mean amplitude (% MVE)		
BB	1.1 ± 1.0	1.4 ± 1.1
TB	0.8 ± 0.6	1.2 ± 0.9
VL	1.2 ± 1.0	1.0 ± 1.0
BF	1.9 ± 1.4	1.5 ± 1.0
Percentage (% time)		
BB	13.6 ± 11.0	21.1 ± 13.9
TB	9.1 ± 8.7	15.9 ± 13.5
VL	11.2 ± 8.7	10.3 ± 7.2
BF	15.3 ± 9.0	17.7 ± 9.9

BB: biceps brachii; TB: triceps brachii; VL: vastus lateralis; BF: biceps femoris; sec: seconds; %: percentage; MVE: maximal voluntary exertion.

*Significantly different from males.

^†^Only old adults.
